# Visible Light Photodegradation of Formaldehyde over TiO_2_ Nanotubes Synthesized via Electrochemical Anodization of Titanium Foil

**DOI:** 10.3390/nano10010128

**Published:** 2020-01-10

**Authors:** Nurul Tasnim Sahrin, Rab Nawaz, Chong Fai Kait, Siew Ling Lee, Mohd Dzul Hakim Wirzal

**Affiliations:** 1 Fundamental and Applied Sciences Department, Universiti Teknologi PETRONAS, Seri Iskandar, Perak 32610, Malaysia; sankhan426@gmail.com; 2 Center for Sustainable Nanomaterials, Ibnu Sina Institute for Scientific and Industrial Research, Universiti Teknologi Malaysia, UTM Johor Bahru, Johor 81310, Malaysia; sllee@ibnusina.utm.my; 3 Chemistry Department, Faculty of Science, Universiti Teknologi Malaysia, UTM Johor Bahru, Johor 81310, Malaysia; 4 Chemical Engineering Department, Universiti Teknologi PETRONAS, Seri Iskandar, Perak 32610, Malaysia; mdzulhakim.wirzal@utp.edu.my

**Keywords:** TiO_2_ nanotubes, anodization, ionic liquid, glycerol, formaldehyde degradation

## Abstract

In this study, a series of TiO_2_ nanotubes (NTs) were synthesized employing electrochemical anodization of titanium foil in an ionic liquid solution containing a mixture of glycerol and choline chloride, acting as electrolyte. The as-synthesized TiO_2_ NTs were calcined at 350, 450, or 550 °C for a 2 h duration to investigate the influence of calcination temperature on NTs formation, morphology, surface properties, crystallinity, and subsequent photocatalytic activity for visible light photodegradation of gaseous formaldehyde (HCHO). Results showed that the calcination temperature has a significant effect on the structure and coverage of TiO_2_ NTs on the surface. Freshly synthesized TiO_2_ NTs showed better-ordered structure compared to calcined samples. There was significant pore rupture with increasing calcination temperature. The transformation from anatase to rutile phase appeared after calcination at 450 °C and the weight fraction of the rutile phase increased from 19% to 36% upon increasing the calcination temperature to 550 °C. The band gaps of the TiO_2_ NTs were in the range from 2.80 to 2.74 eV, shifting the active region of the materials to visible light. The presence of mixed anatase–rutile TiO_2_ phases in the sample calcined at 450 °C showed enhanced photoactivity, which was confirmed by the 21.56 mg∙L^−1^∙g^−1^ removal of gaseous formaldehyde under 120 min of visible light irradiation and displayed enhanced quantum yield, *∅*_HCHO_ of 17%.

## 1. Introduction

Indoor air pollution is one of the major worldwide human health concerns related to volatile organic compounds (VOCs) since it can lead to sick building syndromes such as headache and fatigue. VOCs such as formaldehyde, toluene, and chloroform are the most commonly investigated airborne contaminants [1]. Formaldehyde (HCHO) is considered as one of the most hazardous VOCs because long-term exposure to it causes adverse effects on human health such as eye irritation, breathing difficulties, and skin irritation [2,3]. The World Health Organization (WHO) guidelines for indoor air HCHO concentration is 0.08 ppm. Nielsen et al. [4] reported that the maximum HCHO concentration in a house inhabited by asthmatics in Boston was 162 μg/m^3^ while for homes in Japan the maximum concentration was 58 μg/m^3^, although the value can increase to 220 μg/m^3^ in the summer. Hence, it is crucial to eliminate this chemical substance in order to improve indoor air quality and to comply with stringent environmental regulations.

Several conventional physicochemical methods have been investigated for the removal of HCHO from the air [5,6,7]. However, these methods have several major drawbacks including their incapability to remove HCHO completely from the air, long retention time, and the production of secondary pollutants [8]. Among the advanced oxidation processes (AOPs), photocatalytic technology provides an affordable, economical, and sustainable alternative for the degradation of gaseous formaldehyde [6]. For example, Wu has decomposed 85.6% of formaldehyde over 0.15 g of nitrogen-doped ZnO [9]. Similarly, Chang et al. [10] reported 93.2% formaldehyde degradation over platinum–tin oxide core-shell structured nanoparticles. The major advantage of photocatalytic oxidation is that it offers the possibility of using sunlight as renewable solar energy for photocatalytic degradation of HCHO, thereby making the process green and sustainable. Photocatalytic technology using titanium dioxide (TiO_2_) has been considered as a popular advanced oxidation process [11,12] because of its predominant photocatalytic activity, low toxicity, and high resistance toward corrosion, the low production cost of TiO_2_ [13,14], and its effectiveness in swift degradation of recalcitrant organic compounds and complete mineralization into CO_2_ and water [15,16,17]. Pure or modified TiO_2_ has been used to remove gaseous formaldehyde from airstreams. Zhu and Wu reported 98.3% formaldehyde degradation over Pt-modified TiO_2_ [18]. However, high aggregation tendency and difficulty in separation and recovery of the TiO_2_ are some of the most important limitations of photocatalysis for practical application in air decontamination. To overcome these limitations, the immobilization of TiO_2_ on support medium has become a preferable alternative. For instance, Xu et al. [19] coated TiO_2_ on fabrics for the degradation of HCHO. Their results demonstrated that the TiO_2_ coated on fabrics showed better photocatalytic performance compared to non-coated TiO_2_. Nevertheless, the drawbacks associated with immobilization or coating such as concentrating the target contaminant around the TiO_2_ nanoparticles are also difficult to address convincingly. The other bottleneck for TiO_2_ to be utilized in visible light photocatalysis is its wide bandgap.

These challenges can be overcome by fabricating the nanosized TiO_2_ on some larger particulates and fabricating visible light active TiO_2_. The advent of one-dimensional nanostructures such as TiO_2_ NTs and its fabrication by simple electrochemical anodization of titanium (Ti) foil has greatly eliminated the requirement of a filtration unit or catalyst recovery from the treated air stream. Since then, the decontamination of air with this high surface area and vertically aligned homogeneous TiO_2_ (NTs) has gained increasing research interest. Recently, more research activities were focused on the formation, growth, structural modifications, and applications of TiO_2_ NTs [20]. Several methods exist for the fabrication of TiO_2_ NTs. However, electrochemical anodization offers a simple and more robust way to fabricate TiO_2_ NTs at low cost. Furthermore, TiO_2_ NTs can be grown on titanium substrate producing large surface area materials, hence improving the interaction between the gas and nanostructures while possessing an immobilized characteristic at the same time [15,21]. Although this technique has various advantages, some issues could occur in terms of the reproducibility of the prepared samples. It has been reported that the photocatalytic performance of TiO_2_ NTs strongly depends on its surface morphology [22]. The surface morphology of TiO_2_ NTs are controlled by various experimental parameters such as anodization duration, anodization potential, and electrolyte composition [13]. Calcination temperature of the NTs is another important factor that determines the NTs properties and reaction performance of the photocatalyst [15].

There are many articles that previously reported on the formation of self-organized TiO_2_ NTs by anodization of Ti in an electrolyte containing fluoride ions [12,15,22]. The NTs demonstrated interesting photocatalytic degradation of organic pollutants. For example, Sreekantan et al. [23] reported that uniform and well-aligned TiO_2_ NTs were produced in fluorinated glycerol electrolytes at an anodization voltage of 20 V, which exhibited better photocatalytic activity for degradation of methyl orange. Liang et al. reported more than 90% degradation of 2,3-dicholorphenol over anodic TiO_2_ NT arrays [15]. However, a fluoride-free electrolyte is desired as discussed by Nguyen et al. [24] due to the ease in handling and synthesizing NTs without involving hazardous chemicals, thus allowing the NTs to be grown in a safer manner. There are a few research groups that have investigated the formation of TiO_2_ NTs by anodization process using a chloride-containing electrolyte as an alternative to fluoride-based electrolyte [24,25,26,27]. For instance, Hahn et al. [28] synthesized TiO_2_ and WO_3_ NTs in HClO_4_ and NaClO_4_ electrolytes. Nguyen and co-workers [24] also reported that NTs can be fabricated using NaCl dissolved in either water, ethylene glycol, or glycerol. Their results showed the formation of NTs with a relatively smaller diameter at a faster growth rate compared to those NTs fabricated in a fluoride-based electrolyte. Excellent reviews have been published on the fabrication, modification, and application of TiO_2_ NTs [29,30]. It is evident that TiO_2_ NTs have gained tremendous research attention and there has been an exponential growth in this field.

Heat treatment also plays a crucial role in producing NTs with enhanced photocatalytic activity since high thermal treatment can significantly affect the crystallographic structure of the TiO_2_ phase [31]. Hurum et al. [32] reported that anatase TiO_2_ exhibits lower recombination rates compared to the rutile phase and is regarded as a more photochemically active phase. Other pieces of literature also supported that mixed-phase TiO_2_ exhibited higher photocatalytic activity compared to pure phases alone. Bickley et al. [33] proposed the hypothesis of good photocatalytic activity of mixed-phase TiO_2_ is due to the transfer of electrons from anatase (3.2 eV) to lower energy rutile (3.0 eV) electrons trapping site which serves to reduce the recombination rate of anatase. However, there is no consensus on whether a pure anatase phase or mixed-phase (anatase and rutile) TiO_2_ NTs are the most photochemically active catalyst.

The main aim of the current study is to fabricate visible-light active TiO_2_ NTs for photodegradation of gaseous formaldehyde. TiO_2_ NTs were fabricated by electrochemical anodization of Ti foil in an ionic liquid solution containing a mixture of glycerol and choline chloride (chloride-based electrolyte). The effects of calcination temperature on the properties and photocatalytic activity of the synthesized TiO_2_ NTs were investigated.

## 2. Materials and Methods

### 2.1. Synthesis of Ionic Liquid

**Figure 1:**
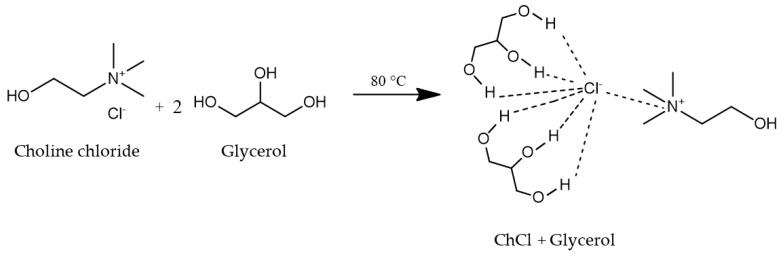
Chemical structure of choline chloride and glycerol [36].

Choline chloride (ChCl, 98%, Sigma Aldrich, Darmstadt, Germany), and glycerol (C_3_H_8_O_3_, 98%, Fischer Scientific, NH, USA) were mixed in a molar ratio of 1:2 and heated to 80 °C for 30 min to form a colorless ionic liquid [34,35]. It was used as the electrolyte solution for the anodization process of Ti foil to form TiO_2_ NTs. The chemical structure of the mixture of ChCl and glycerol is shown in Figure 1.

### 2.2. Synthesis of TiO_2_ Nanotubes

**Figure 2:**
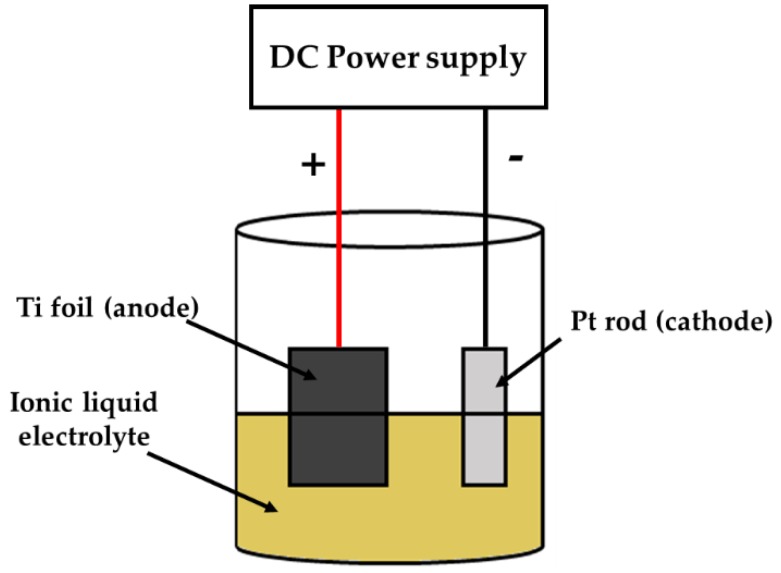
Schematic diagram of the anodization setup.

Technical grade Ti foil (Titanium Ti Gr5/Tc4 Grade 5 ASTM B265 Thin Plate Sheet, Nanjing, Jiangsu, China) with 0.1 mm thickness was cut into 2 cm × 1 cm squares which were used as the substrates for anodization process. The Ti substrates were ultrasonically cleaned in acetone for 10 min, followed by thorough rinsing with deionized (DI) water, and dried in air prior to anodization. Figure 2 shows the experimental set up for the fabrication of TiO_2_ NTs. The anodization process was conducted in an electrochemical set up consisting of Ti substrate as the anode and platinum rod as the cathode. The distance between cathode and anode was fixed at 2.5 cm. The electrodes were submerged in 35 mL of ionic liquid electrolyte solution and the experiment was conducted for 1 h at a constant voltage of 20 V (DC power supply) and under ambient condition. After anodization, the as-synthesized samples were removed immediately from the electrolyte solution and rinsed with distilled water. The samples were air-dried in ambient atmosphere. The as-synthesized TiO_2_ NTs (denoted as TiO_2_) were calcined at 350, 450, or 550 °C for 2 h with a heating rate of 5 °C/min in static air. The calcined TiO_2_ NTs were labeled as T_TiO_2_, where ‘T’ refers to the calcination temperature in °C. For example, 350_TiO_2_ represents as-synthesized TiO_2_ NTs calcined at 350 °C.

### 2.3. TiO_2_ Nanotubes Characterization

The prepared TiO_2_ NTs were characterized using Field emission scanning electron microscopy (FESEM) from Carl Zeiss instrument (SUPRA 55VP, Oberkochen, Germany) to investigate the surface morphology. The FESEM images were captured at 50 kX at an acceleration voltage of 20 kV. The crystalline structure and phase composition of TiO_2_ NTs were determined using X-ray diffractometer (PANalytical X’Pert^3^ Powder, AA Almelo, Almelo, The Netherlands) with Cu Kα radiation (40 kV, 40 mA) at a 2θ angle of 10° to 80° with the step size of 0.01°. The weight fractions of the anatase and rutile phases of the NTs, calcined at various temperatures, were estimated using Equation (1) [15,23] based on the relative intensities of the most dominant peaks for rutile (*I_R_*) and anatase (*I_A_*) at (110) and (101) planes, respectively:

(1) $$f_{r}=\frac{1.26I_{R}}{I_{A}}+1.26\left(I_{R}\right)$$

The average crystallite sizes of the TiO_2_ NTs were determined from the characteristic diffraction peaks matching the (101) plane of anatase TiO_2_ at 2θ = 25° and the rutile crystallite was calculated from the rutile peak located at 2θ = 27° using Scherrer’s formula as shown in Equation (2):

(2) $$D=\frac{K\lambda }{\beta \mathrm{Cos}\theta }$$

where K is the Scherrer’s constant (0.9), λ is the X-ray wavelength (0.15418 nm), β is the full width at half maximum of the selected diffraction peak (in radian), and θ is the Bragg’s angle.

The TiO_2_ NTs were further analyzed using XPS from Thermo-Fischer (K-alpha, Madison, WI, USA). Al Kα (1486.60 eV) was used as an X-ray excitation source with C1s correction at 285.73 eV for calibration. The Ti2p and O1s XPS signals were deconvoluted using Gaussian curve fitting. Photoluminescence (PL) spectra of the TiO_2_ NTs were recorded using Horiba LabRam HR Evolution spectrometer (Minamiku Kyoto, Japan) at room temperature operated at 325 nm excitation light equipped with He-Cd laser. The PL spectra were recorded in the range from 350 nm to 700 nm. The bandgap values were estimated using Planck’s expression as shown in Equation (3) below:

(3) $$E=\frac{hc}{\lambda }$$

where *E* is photon energy (eV), h is Planck’s constant (6.626 × 10^−34^ J.s), c is speed of light constant (3.00 × 10^8^ ms^−1^), and λ is wavelength (m).

### 2.4. Photodegradation of Formaldehyde

**Figure 3:**
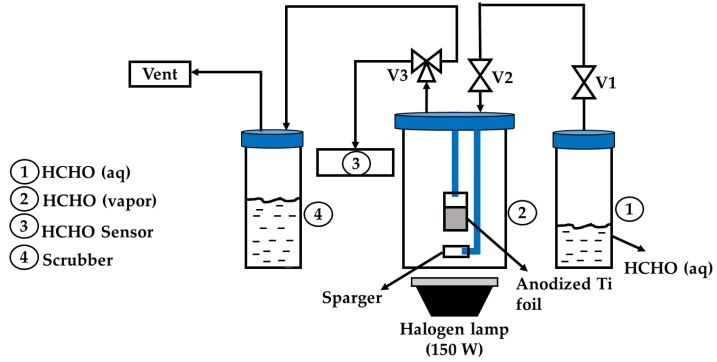
Photoreactor system for formaldehyde (HCHO) photodegradation.

The photocatalytic experiments for photodegradation of HCHO were carried out in batch mode. The photoreactor system for HCHO photodegradation under visible light irradiation is shown in Figure 3. The anodized Ti foil containing TiO_2_ NTs was positioned inside a 250 mL quartz photoreactor with a holder. HCHO (37% in aqueous solution, Sigma Aldrich, Darmstadt, Germany) of 3.22 ppm was added inside a tightly sealed stainless-steel container (1). The quartz photoreactor (2) was filled with HCHO vapor by diffusion when V1 and V2 valves were opened. After 30 min equilibration in the dark, the quartz photoreactor was irradiated with a halogen lamp (150 W) as the light source, which mainly consists of a visible light region, and was positioned 7 cm beneath the photoreactor. The intensity and wavelength of the light was 1379.67 W/m^2^ and 400–750 nm, respectively. The photodegradation of HCHO was monitored by sampling at 30 min interval for 150 min. A digital formaldehyde sensing meter (Hal Tech, Wetherill Parl, NSW Australia) (3) was connected to V3 to measure the HCHO vapor concentration. The working range of the sensor is up to 10 ppm. During the photoreaction, the temperature of the photoreactor was maintained at 25 ± 1 °C using a cooling fan. At the end of the reaction, the remaining gas flows through scrubber (4) containing H_2_O, which acts as the scrubber for HCHO.

The photodegradation performance, *X* was monitored and calculated using Equation (4):

(4) $$X\left(\%\right)=\frac{C_{0}-C_{t}}{C_{0}}\times 100\%$$

where *X*% denotes the percentage of HCHO removal, *C*_0_ is the initial concentration of HCHO, and *C_t_* represents the concentration of formaldehyde at sampling time, *t*.

## 3. Results and Discussion

### 3.1. Characterization of the TiO_2_ Nanotubes

#### 3.1.1. Surface Morphology

**Figure 4:**
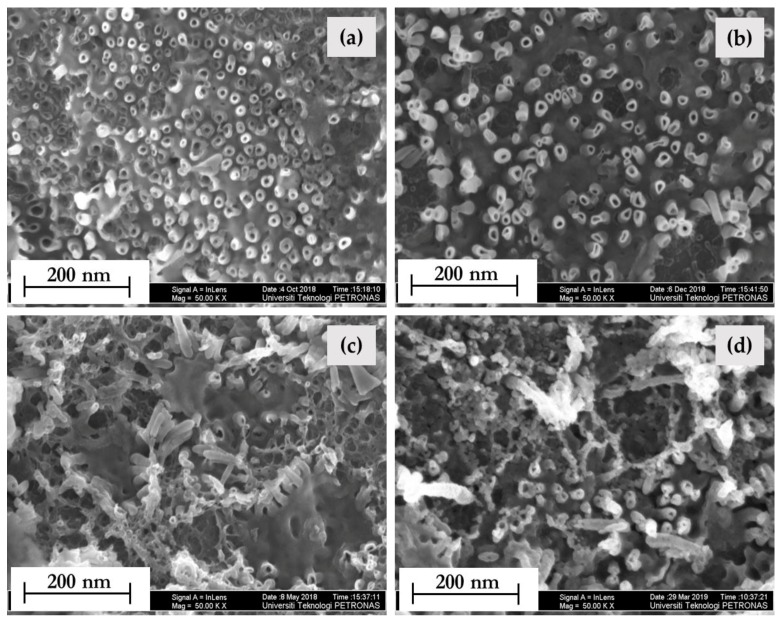
Field emission scanning electron microscopy **(**FESEM) images of anodized Ti foil for (**a**) uncalcined and after calcination at (**b**) 350 °C, (**c**) 450 °C, and (**d**) 550 °C at 50 kX magnification.

Figure 4 shows the effect of calcination temperature on the morphology of the synthesized TiO_2_ NTs. Figure 4a of as-synthesized TiO_2_ NTs showed discrete and irregular shaped TiO_2_ NTs formed covering the surface of the Ti foil when no heat treatment was introduced. It can be observed that almost the whole surface is covered by TiO_2_ NTs and is denser than other samples that were calcined at different temperatures. As for the TiO_2_ NTs calcined at 350, 450, and 550 °C, obvious changes can be seen from Figure 4b–d. The tubular structure was distorted, and a more severe pore rupture and disintegration of the surface was observed upon increasing calcination temperature to 550 °C. It is quite evident that the NTs after being calcined apparently had a poorly ordered structure, which can be attributed to the increase in internal stress with the shrinkage of thin-film during the calcination process [37] that destroys the NTs structure. This may also be ascribed to destruction and coalescence at the top of the NTs walls [38]. Furthermore, with increasing calcination temperature, a large area of the surface was covered by isolated NTs indicating that the better-ordered structure was destroyed to a great extent, which can be due to the phase transformation from anatase to rutile [37]. In fact, the phase transition from anatase to rutile was confirmed by X-ray diffraction analysis as discussed in Section 3.1.4.

It is important to note that chloride-containing electrolyte (ChCl) was employed for the fabrication of TiO_2_ NTs in the present study as opposed to previous works where fluoride-based electrolytes were predominantly used for the fabrication of NTs. Previous reports by Hahn et al. [28] and Ng et al. [39] provides evidence that chloride ion in an organic electrolyte can be used to grow TiO_2_ NTs efficiently. In addition, the viscosity of organic-based electrolytes can influence the diffusion of ionic species, resulting in the altering of the morphology of NTs. Bervian et al. [40] found that TiO_2_ NTs anodized in a glycerol-based electrolyte displayed higher mobility of ionic species, thus resulted in increased growth rate.

The mechanism of TiO_2_ NTs formation on the surface of Ti foil can be explained using Equations (4)–(7). The growth of oxide on the surface of Ti foil is from the interaction between cations (Ti^4+^) and O^2^^−^ species formed in the organic electrolyte [41]. During the early stage of the anodization process, a TiO_2_ layer was grown on the surface of the Ti foil after a fixed potential (20 V) was applied (Equation (6)). According to reported principle, TiO_2_ growth was based on three key processes [42]. On the surface of Ti substrate, there was the formation of a TiO_2_ layer, which can be expressed by Equation (8) [42,43,44]:

Process 1: Field-assisted oxidation at metal/oxide interface

Ti → Ti^4+^ + 4e^−^ (5)

Ti^4+^+ 2O^2^^−^ → TiO_2_ (6)

Ti(OH)_4_ → TiO_2_ + 2H_2_O (7)

2Ti + 2H_2_O → 2TiO_2_ + 4e^−^ + 4H^+^ (8)

After that, Cl^−^ ions randomly attacked TiO_2_ and TiO_2_ started dissolving due to electrochemical etching leading to the formation of pits and a thick layer of TiO_2_ due to migration of oxide ions. NTs were then grown and elongated inside of the pits and the oxide layer releases periodically until the complete transformation of Ti metal to TiO_2_. The negatively charged ions within the electrolyte, particularly OH^−^ and Cl^−^, moved toward Ti. The OH^−^ would be responsible for the formation of TiO_2_. In our case, the OH^−^ is most probably coming from the water content that is present in the mixture of choline chloride and glycerol. In fact, the water content in the mixture was confirmed from water content analysis using Karl Fischer assay and was found to be 0.5%. A sample of the mixture of ChCl and glycerol along with the reagent (CombiTitrant) were introduced into a titration cell and dissolved. The reagent was released by the induction of an electrical current and the amount of current required to convert the water is the determinant of the amount of moisture present in the sample. Karl Fischer assay was used because it has the capability to measure the moisture or water content as low as 200 µg. Meanwhile, randomly attacked Cl^−^ ions etched the Ti metal, which cause dissolution of Ti as [TiCl_6_]^2^^−^ for nanotubes formation [43,45,46,47]. The formation of TiO_2_ nanotubes under the influence of Cl^−^ is given in Equations (9) and (10) [48]:

Process 2: Field-assisted dissolution at oxide/electrolyte interface at tube bottom

Ti^4+^ + 6Cl^−^ → [TiCl_6_]^2−^ (9)

Process 3: Chemical dissolution/etching of the fabricated tubes at the tubes top

TiO_2_ + 4H^+^ +6Cl^−^ → [TiCl_6_]^2−^ + 2H_2_O (10)

**Figure 5:**
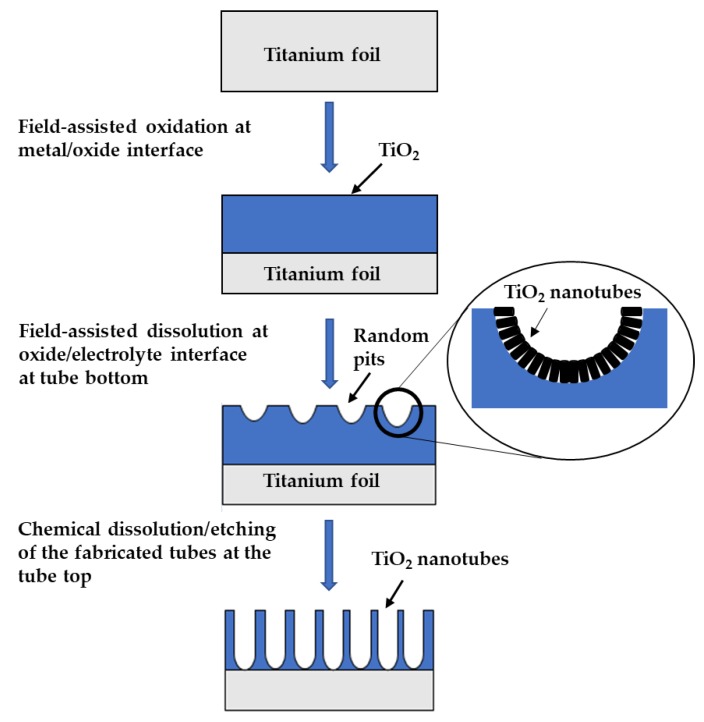
Illustration of the formation TiO_2_ nanotubes adapted from Reference [39].

To further understand the mechanism of nanotubes formation, the anodization reaction could be represented by the simplified diagram shown in Figure 5 [39]:

#### 3.1.2. XPS Analysis

**Figure 6:**
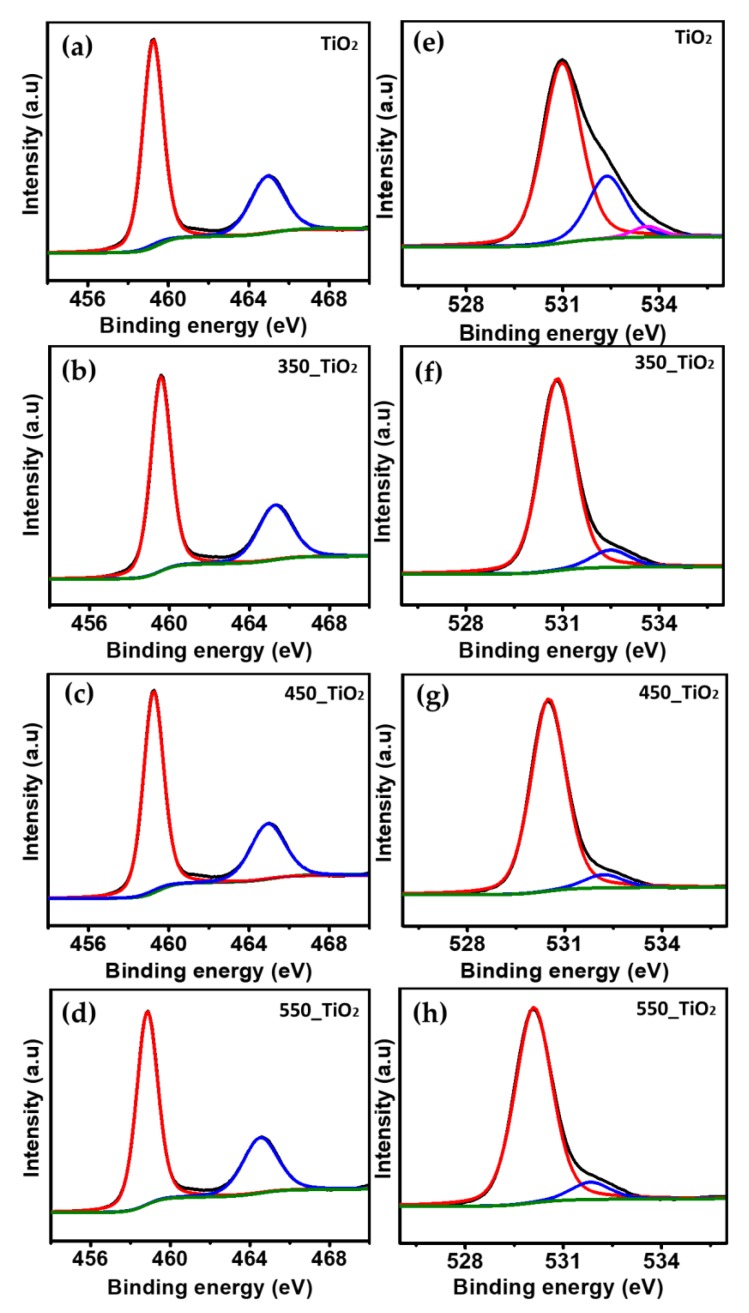
XPS spectra of (**a**–**d**) Ti2p and (**e**–**h**) O1s of TiO_2_ nanotubes (NTs) calcined at various temperatures.

XPS analysis was performed in order to obtain better insight into the surface properties and nature of the chemical bonding in the synthesized TiO_2_ NTs. Figure 6 shows the high-resolution deconvoluted Ti2p and O1s spectra of the TiO_2_ and TiO_2_ NTs calcined at different temperatures. For the TiO_2_ sample, the binding energies (BE) of Ti2p_3/2_ and Ti2p_1/2_ were located at 459.4 and 465.2 eV, respectively, which are the characteristic features of anatase. The calculated difference in BE of Ti2p_3/2_ and Ti2p_1/2_ (∆BE = BE Ti2p_3/2_ − Ti2p_1/2_) is equaled to 5.8 eV, which are characteristic to Ti^4+^–O bonds in TiO_2_ [45,46]. Similar Ti2p_3/2_ and Ti2p_1/2_ peak positions were observed for the 350_TiO_2_ NTs. However, the Ti2p_3/2_ and Ti2p_1/2_ peaks of the sample calcined at 450 °C exhibit a negative shift of 0.1 eV and the peaks are now located at 459.3 eV and 464.8 eV. Compared to the TiO_2_ sample, the 550_TiO_2_ NTs shows a negative shift 0.6 eV of Ti2p_3/2_ and Ti2p_1/2_ and the peaks are now centered at 458.8 and 464.5 eV, respectively.

The shift in the position of Ti2p_3/2_ and Ti2p_1/2_ peaks indicated the influence of calcination temperature on the electronic state of the Ti element; most probably, the Ti^4+^ is reduced partially due to the loss of oxygen as the heat treatment temperature was increased from 350 to 550 °C [47]. According to previous reports on TiO_2_ NTs, a negative shift in BE is associated with the additional screening of extra electrons in the crystal field with an increase in calcination temperature [49]. However, the ΔBE value between Tip_1/2_ and Tip_3/2_ for samples 350_TiO_2_, 450_TiO_2_, and 550_TiO_2_ were 5.7, 5.6, and 5.7 eV, respectively, which are typical of the existence of Ti^4+^ on the surface of TiO_2_ lattice [45].

The major peaks of O1s with BE regions of 530.8–530.0 eV refer to Ti^4+^–O in TiO_2_ [37,50]. The rest of the two peaks at BE region 532.1–531.2 eV and 533.7–532.2 eV were associated with adsorbed oxygen and OH group, respectively [51,52]. It was reported that the OH group gradually decreased by increasing the calcination temperature, which was suggested due to the chemical reaction that took place on the surface of TiO_2_ during the heat treatment process, as shown in Equation (11) [50]:

Ti–OH + HO–Ti → Ti–O–Ti + H_2_O (11)

#### 3.1.3. Optical Properties

**Figure 7:**
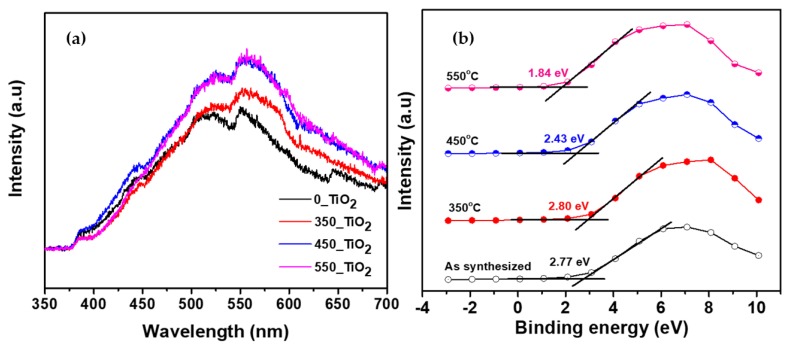
The effect of calcination temperatures on (**a**) the PL spectra and (**b**) the valance band positions of TiO_2_ NTs.

The photocatalytic activity of TiO_2_ depends on the duration of the separation between the excited electrons and holes on its surface. Therefore, the photoluminescence (PL) spectra of the TiO_2_ NTs were measured to investigate the effect of calcination temperature on the optical properties and behavior of electron-hole recombination. The emission spectra of the as-synthesized and calcined TiO_2_ NTs in the wavelength range of 350–700 nm, with excitation at 325 nm, are shown in Figure 7a. The main emission peaks of all the TiO_2_ NTs appeared at 386 nm (3.21 eV), 445 nm (2.80 eV), 555 nm (2.23 eV), and 596 nm (2.08 eV). The peak at 386 nm was attributed to the bandgap transition, corresponding to the bandgap energy of anatase. The samples 0_TiO_2_, 350_TiO_2_, 450_TiO_2_, and 550_TiO_2_ showed the emission peaks at 442 nm, 445 nm, 448 nm, and 452 nm, respectively, which can be attributed to the band-edge free excitation. These wavelengths (nm) were converted to energy (eV) according to Pishkar et al. [53] and the values are given in Table 1. From the inspection of Table 1, it appears that the bandgap gap energy decreased with increasing calcination temperature and the results are consistent with the results reported by Mioduska et al. [54] that the calcination temperature can influence the energy bandgap. It is suggested that the increase in temperature can cause an incremental increase in the absorption coefficient due to the increase of defects sites. Electron-hole pairs are produced through photon absorption, generating a field that could change the optical attributes and electronic structure of the product [55].

The PL peak intensities of the as-synthesized TiO_2_ NTs showed a significant decrease compared to those calcined at 350, 450, and 550 °C. Furthermore, the intensity variations suggest that the PL spectrum of TiO_2_ NTs with larger crystallite size is dominated by a radiative recombination of electrons via intrinsic defects states. The blue emission is visible due to the available surface energy that induces quantum confinement effect and also due to the presence of oxygen vacancies defects on the surface of TiO_2_ [56].

The bandgap values obtained from the PL spectrum are shown in Table 1. No significant effect of calcination temperature on the bandgap values of the NTs was observed in the present study. However, a variation in calcination temperatures showed a significant effect on the valence band positions of the TiO_2_ NTs, as shown by the valence band (VB) XPS spectra in Figure 7b. The valence band of the as-synthesized NTs was located at 2.77 eV, while the valence band positions were shifted to 2.80, 2.43, and 1.84 eV with the increase in calcination temperature to 350, 450, and 550 °C, respectively. Pishkar et al. [53] and Ghows and Entezari [57] reported in their findings that bandgap values calculated from the PL spectrum are in good agreement with their diffuse reflectance spectra (DRS) results. It can be observed that the PL peak intensity gradually increases with increasing calcination temperature. Yu et al. and Sang et al. [58,59] reported that low PL intensity suggests a low recombination rate of excited charges on the surface of the photocatalyst. As expected, the high crystallinity and low number of defects sites results in a lower PL intensity, which is consistent with XRD patterns. However, it should be noted that the sample calcined at 350 °C is highly crystalline as compared to the uncalcined sample. but the slight increase in PL intensity of the 350_TiO_2_ can be due to the presence of random cracks on the surface of Ti foil [58]. The bandgap energy decreases with increasing calcination temperature, which reflects anatase to rutile phase alteration as inferred from the XRD analysis. A previous study also reported that, usually, PL intensity is proportional to the amount of produced hydroxyl radicals that are needed to enhance photocatalytic performance [38].

#### 3.1.4. XRD Analysis

**Figure 8:**
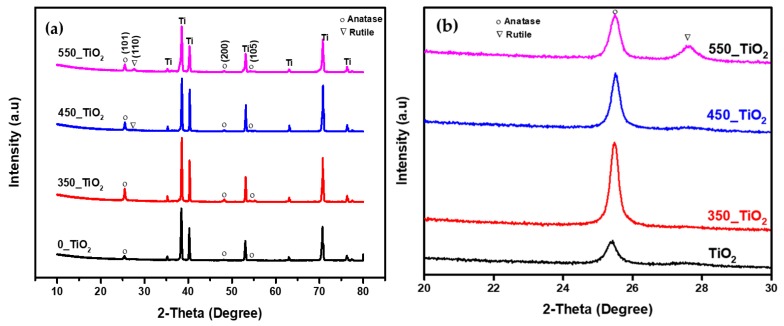
XRD patterns for TiO_2_ calcined at different calcination temperature (**a**,**b**) magnified XRD patterns from 20° to 30° (2θ).

The mechanism of phase transformation is important in order to control the nanostructure and material properties. The crystallite size and crystallinity, as well as phase structure and composition, play an important role in the photocatalytic activity of TiO_2_. Figure 8a displays the XRD patterns of the synthesized TiO_2_ NTs. TiO_2_, 350_TiO_2_, and 450_TiO_2_ patterns congruously show characteristic diffraction peaks at 2θ = 25.2°, 48.1°, and 54.1° corresponding to 101, 200, and 105 planes of the anatase phase, respectively. It can be observed for the peak at 2θ = 25.2° that its intensity increased at 350 °C calcination temperature but decreased when higher heat treatment was introduced. Compared to the other samples, TiO_2_ NTs calcined at 450 °C exhibited the rutile phase with a peak at 2θ = 27.5°, as shown in Figure 8b. The weight fraction of the rutile peak was 19%. The sample calcined at 550 °C (550_TiO_2_ NTs) exhibited one additional peak of 2θ = 69.9° corresponding to 220 planes of rutile. With this additional rutile peak, the weight fraction was increased to 36%. The results suggest that the anatase to rutile phase transformation occurs at a higher calcination temperature. It has been reported that the anatase crystal phase can be induced by thermal treatment starting from 350 °C and the shift of the diffraction peaks to a higher angle could be attributed to the difference in particle size, impurities, and synthesis methods [50].

The average crystallite sizes of the TiO_2_ NTs calcined at different temperatures are listed in Table 1. The TiO_2_ NTs calcined at 350 °C showed the highest crystallite size (60.50 nm) compared to other samples. It should be noted that the calculated crystallite size was larger than the inner tube diameter of 350_TiO_2_ sample, which can be due to the inhomogeneous distribution of TiO_2_ NTs [60]. There is a decreasing trend of the inner tube diameter in the temperature range 450–550 °C for the average size of the anatase grains (from 48.84 to 25.05 nm). This suggests that in this range of temperature, some parts of anatase grains started to transform into rutile TiO_2_ [61]. Anatase to rutile transformation happens by coarsening, so rutile crystallite size is expected to be bigger than anatase. In the present study, the crystallite size (13.93 nm) of rutile was smaller than the crystallite size of anatase (44.36 nm) at a calcination temperature of 450 °C. One of the possible reasons for this is that the values of crystallite sizes reported here are only relative because the contribution of the strain on peak broadening has not been taken into consideration in the calculation using the Scherrer Equation. The other reason could be the appearance of rutile at a lower temperature (450 °C) in our prepared samples as compared to previous reports, where they reported the transformation of anatase to rutile to have occurred at 600 °C.

However, the crystallite size of rutile rose up to 27.85 nm when the calcination temperature was increased to 550 °C [61]. Generally, the rutile phase appears at higher temperature ca. 500 °C. For instance, Low et al. studied the crystalline behavior of pure and chromium-doped TiO_2_ nanotubes, where they reported the transformation of anatase to rutile to have occurred at 600 °C [62]. On inspection of Table 1, it is evident that the crystallite size of pure TiO_2_ NTs was smaller compared to the calcined TiO_2_ NTs samples. Initially, the crystallite size of anatase rose up to 60.50 nm when the calcination temperature was increased to 350 °C. However, the anatase crystallites size began to decrease when the calcination temperature was raised to 450 and 550 °C. This could be due to the fact greater anatase phase crystallites first turn into the rutile phase during the phase transition, which results in the decrease of average crystallite size of the anatase.

The dominant peak at 2θ = 25.2° showed the highest intensity when calcined at 350 °C. However, a higher calcination temperature led to a decreased intensity of the anatase peak. This implies that the crystallinity of the TiO_2_ NTs decreased with increasing calcination temperature.

### 3.2. Photocatalytic Activity

**Figure 9:**
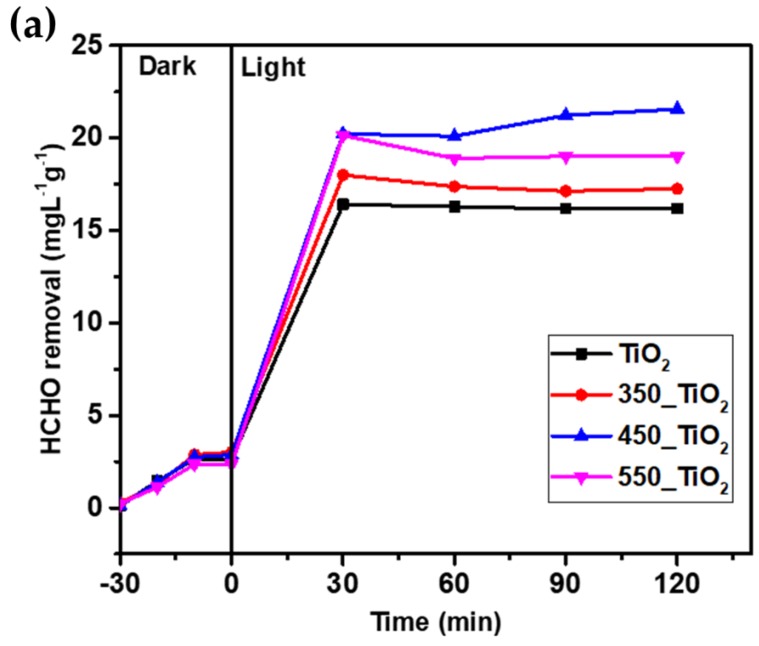

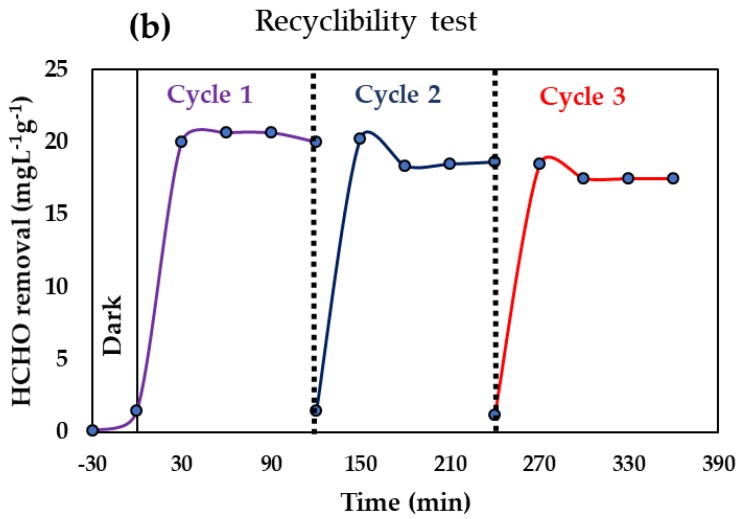
(**a**) Effect of calcination temperatures on the photodegradation of formaldehyde. Experimental conditions: 3.22 ppm initial formaldehyde concentration, 25 °C, 150 W, and (**b**) recycling results of 450_TiO_2_ HCHO removal.

The photocatalytic activities of the fabricated TiO_2_ NTs were examined for the removal of gaseous HCHO. Figure 9a shows the removal of HCHO using four different type TiO_2_ NTs: as-synthesized NTs, and NTs calcined at 350 °C, 450 °C, and 550 °C under visible light irradiation. Without light (adsorption equilibrium), almost 9% of HCHO was removed by TiO_2_ NTs. A control experiment has been done for blank where no reaction has been observed. A noticeable improvement of HCHO removal was observed in the presence of light suggesting significant photocatalytic activity of the fabricated TiO_2_ NTs. The higher HCHO removal efficiency (62%) was obtained by TiO_2_ NTs calcined at 450 °C. The higher efficiency of 450_TiO_2_ NTs can be attributed to its mixed crystal composition of anatase and rutile, where the rutile phase is dispersed into the anatase phase, resulting in an improvement of electronic interactions between the anatase and rutile phases. In other words, it is believed that the lower bandgap rutile phase absorbs photons and formed electron-hole pairs while the anatase phase traps the electrons. Thus, reducing the recombination of electron and allowing the hole to transfer to the surface to react. It has been reported that the number of photons being in contact with the surface of the photocatalyst actually controls the degradation reaction [63]. The latter is an indication that the reaction takes place only in the adsorbed phase of the photocatalyst. Although uncalcined TiO_2_ NTs exhibits anatase phase, its photocatalytic activity was lower (46%) than the calcined samples. Nevertheless, the HCHO removal efficiency in the current study is higher as compared to a previous study, where they only removed 40% of HCHO from air using TiO_2_ immobilized on a low melting point polymer (TiO_2_@LMPET) [51].

The synthesized TiO_2_ NTs showed dissimilar HCHO removal concentration. This can be due to the structural dissimilarity and differently sized inner tube diameter. The uncalcined sample and the NTs samples calcined at 350 and 550 showed 16.17, 17.25, and 19.01 mg∙L^−1^∙g^−1^ HCHO removal concentration, respectively. The HCHO removal concentration of 450_TiO_2_ NTs was 21.56 mg∙L^−1^∙g^−1^, which is highest among all the samples. The results demonstrate that the HCHO removal concentration shows a significant increase when increasing the calcination temperature to 450 °C. However, above 450 °C, the photocatalytic removal concentration of HCHO declines. TiO_2_ NTs sample calcined at 450 °C achieved the highest degree of HCHO degradation may be ascribed due to the optimum crystallinity of anatase and rutile phase developed at this temperature [15]. It is well known that Degussa P25-TiO_2_ is often used as the benchmark photocatalyst due to its superior photocatalytic activity, which contains 80% anatase and 20% rutile phases [64]. This implies that the presence of optimum anatase to rutile mass ratio is also beneficial to increase the photocatalytic activity [65]. Furthermore, Razali et al. stated that anatase fraction can provide a high amount of oxygen vacancy sites that could contribute to the production of active radicals. There is a direct correlation between the adsorption of organic pollutants and surface coverage of the TiO_2_ photocatalyst [66]. Meanwhile, the quantum yield of HCHO degradation, *∅*_HCHO_ for TiO_2_ and 350_TiO_2_, was 0.95 and 0.98, respectively. Most importantly, it was found that it further increased the calcination temperature to 450 and 550 °C led to the enhancement of *∅*_HCHO_ by almost 17% (*∅*_HCHO_ = 1.14 and 1.28, respectively). Quantum yield is defined as the light efficiency of processes, the ratio of the reaction rate to the absorption rate of photons [67,68]. Significantly, the fabrication of TiO_2_ NTs and application for the removal of gaseous HCHO from air have some other implications as well. The fabrication of TiO_2_ NTs can eliminate the immobilization of TiO_2_ on the support medium. Furthermore, the TiO_2_ NTs display a promising future in virtue of the mild operation conditions required for their use and suitability for large-scale fabrication, which could be employed in indoor pollution control technology.

The stability of the photocatalyst is an important consideration for industrial-scale applications in indoor air pollution abatement. To demonstrate the stability of the synthesized TiO_2_ NTs photocatalyst, based on its high performance, 450_TiO_2_ was reused for the recycling tests for three consecutive runs due to its higher photocatalytic performance as. The results of the recyclability tests are shown in Figure 9b. The photocatalytic activity reduced from 20.00 mg∙L^−1^∙g^−1^ to 17.50 mg∙L^−1^∙g^−1^ after the three consecutive runs, and the photocatalytic activity was well retained. The results indicate that synthesized TiO_2_ NTs are very stable under the employed reaction conditions.

Based on previously published reports, HCHO was adsorbed onto the surface of TiO_2_ NTs [7,69,70]. In the below reaction pathway, it is possible that each oxidation step may proceed on the photocatalyst surface either by direct reaction of the adsorbed organic molecules with valence band holes (h_VB_) or by an indirect reaction path through hydroxyl radical attack [30]. For pure anatase TiO_2_, when irradiated with ultraviolet light having photon energy higher than its bandgap, the electron in the conduction band can be photo-excited and transferred to the molecular oxygen provided that the gas inside the photoreactor was air-producing reactive oxygen radicals ^•^O_2_^−^(Equation (13)), where O_2_ acts as an oxidizing agent that can degrade HCHO molecules. In **•**OH, the radical-mediated path implies, first of all, the electrons were excited from the valance band to the conduction band of TiO_2_ NTs and leaving holes behind (h_VB_) in Equation (12) [30]. The h_VB_ reacts with adsorbed water, producing protons and reactive hydroxyl radicals in Equation (14). Photocatalytic oxidation of HCHO results from Equation (15), i.e., from the interaction of **•**OH radicals produced by the reaction of water with excited charge carrier (h_VB_), accumulated on the TiO_2_ NTs under visible light irradiation. It is important to note that Equation (15) cannot proceed via a direct hole-mediated path, but can only proceed through the attack of hydroxyl radicals because an extra oxygen atom is required to transform HCHO into HCOOH, which can only be provided indirectly by water through **•**OH radicals.

TiO_2_ + h_VB_ → e^−^ + h^+^ (12)

e^−^ + O_2_ → •O_2_^−^ (13)

h^+^ + H_2_O → **•**OH + H^+^ (14)

•OH + HCHO → H_2_O + •CHO (15)

**•**CHO + **•**OH → HCOOH (16)

HCOOH + **•**OH → **•**COOH + H^+^ (17)

•COOH + •OH → CO_2_ +H_2_O (18)

## 4. Conclusions

Mixed anatase–rutile phase TiO_2_ nanotubes were successfully fabricated via electrochemical anodization of titanium foil in this study. The TiO_2_ nanotubes with different crystallinities, lower bandgap, and varying crystal composition including pure anatase and mixed-phase anatase–rutile were grown on Ti foil using a mixed electrolyte containing glycerol and choline chloride at a constant voltage of 20 V for 1 h. The effect of calcination temperature on the properties of the TiO_2_ nanotubes was studied. The calcination temperature greatly affects the crystallization, crystalline structure, phase composition, and surface morphology. At a low temperature, pure anatase phase TiO_2_ nanotubes were obtained. The phase transformation from anatase to rutile occurs at 450 °C to 550 °C. High calcination temperature led to the distortion of the TiO_2_ nanotubes. The bandgap of the fabricated TiO_2_ nanotubes was reduced to 2.74 eV from 3.20 eV of standard anatase TiO_2_. Interestingly, calcination at 450 °C led to enhanced photocatalytic activity of the TiO_2_ nanotube as well as *∅*_HCHO_ by 17%, which may be due to the synergistic effect of the mixed anatase–rutile phase. Almost 21.56 mg∙L^−1^∙g^−1^ of gaseous formaldehyde was removed within 120 min of visible light reaction.

## Figures and Tables

**Table 1:** Crystallite size, inner tube diameter, and bandgap of the TiO_2_ photocatalyst from different characterization methods.

Photocatalyst	Average Crystallite Size (nm)		Band Gap Calculated from PL (eV)	Valance Band Positions (eV)
	Anatase	Rutile		
TiO_2_	30.31	-	2.80	2.77
350_TiO_2_	60.50	-	2.76	2.80
450_TiO_2_	44.36	13.93	2.79	2.43
550_TiO_2_	33.27	27.85	2.74	1.84

## References

[1] Huang Y., Ho S.S.H., Lu Y., Niu R., Xu L., Cao J., Lee S. (2016). Removal of Indoor Volatile Organic Compounds via Photocatalytic Oxidation: A Short Review and Prospect. Molecules.

[2] Li W., Liang R., Hu A., Huang Z., Zhou Y.N. (2014). Generation of Oxygen Vacancies in Visible Light Activated One-Dimensional Iodine TiO_2_ Photocatalysts. RSC Adv..

[3] Lin L., Chai Y., Zhao B., Wei W., He D., He B., Tang Q. (2013). Photocatalytic Oxidation for Degradation of VOCs. OJIC.

[4] Nielsen G.D., Larsen S.T., Wolkoff P. (2017). Re-evaluation of the WHO (2010) Formaldehyde Indoor Air Quality Guideline for Cancer Risk Assessment. Arch. Toxicol..

[5] Yan Z., Xu Z., Yu J., Jaroniec M. (2016). Enhanced Formaldehyde Oxidation on CeO_2_/AlOOH-supported Pt Catalyst at Room Temperature. Appl. Catal. B Environ..

[6] Tasbihi M., Bendyna J.K., Notten P.H.L., Hintzen H.T. (2015). A Short Review on Photocatalytic Degradation of Formaldehyde. J. Nanosci. Nanotechnol..

[7] Wang J., Wang J., Wu X., Zhang G. (2017). Pt-TiO_2_ Microspheres with Exposed {001} Facets for Degradation of Formaldehyde in Air: Formation Mechanism and Enhanced Visible Light Photocatalytic Activity. Mater. Res. Bull..

[8] Zhang W., Song N., Guan L., Li F., Yao M. (2016). Photocatalytic Degradation of Formaldehyde by Nanostructured TiO_2_ Composite Films. J. Exp. Nanosci..

[9] Wu C. (2014). Facile One-Step Synthesis of N-doped ZnO Micropolyhedrons for Efficient Photocatalytic Degradation of Formaldehyde Under Visible-Light Irradiation. Appl. Surf. Sci..

[10] Chang Y.C., Yan C.Y., Wu R.J. (2014). Preparation of Pt@SnO_2_ Core-Shell Nanoparticles for Photocatalytic Degradation of Formaldehyde. J. Chin. Chem. Soc..

[11] Nazari M., Golestani-Fard F., Bayati R., Eftekhari-Yekta B. (2015). Enhanced Photocatalytic Activity in Anodized WO_3_-loaded TiO_2_ Nanotubes. Superlattices Microstruct..

[12] Regonini D., Clemens F.J. (2015). Anodized TiO_2_ Nanotubes: Effect of Anodizing Time on Film Length, Morphology and Photoelectrochemical Properties. Mater. Lett..

[13] Qin L., Chen Q., Lan R., Jiang R., Quan X., Xu B., Zhang F., Jia Y. (2015). Effect of Anodization Parameters on Morphology and Photocatalysis Properties of TiO_2_ Nanotube Arrays. J. Mater. Sci. Technol..

[14] Puga A.V. (2016). Photocatalytic Production of Hydrogen from Biomass-Derived Feedstocks. Coord. Chem. Rev..

[15] Liang H., Li X. (2009). Effects of Structure of Anodic TiO_2_ Nanotube Arrays on Photocatalytic Activity for the Degradation of 2,3-Dichlorophenol in Aqueous Solution. J. Hazard. Mater..

[16] Hoffmann M.R., Martin S.T., Choi W., Bahnemann D.W. (1995). Environmental Applications of Semiconductor Photocatalysis. Chem. Rev..

[17] Fagan R., McCormack D.E., Dionysiou D.D., Pillai S.C. (2016). A Review of Solar and Visible Light Active TiO_2_ Photocatalysis for Treating Bacteria, Cyanotoxins and Contaminants of Emerging Concern. Mater. Sci. Semicond. Proc..

[18] Zhu Z., Wu R.J. (2015). The Degradation of Formaldehyde Using a Pt@TiO_2_ Nanoparticles in Presence of Visible Light Irradiation at Room Temperature. J. Taiwan Inst. Chem. Eng..

[19] Xu S., Lu W., Chen S., Xu Z., Xu T., Sharma V.K., Chen W. (2019). Colored TiO_2_ Composites Embedded on Fabrics as Photocatalysts: Decontamination of Formaldehyde and Deactivation of Bacteria in Water and Air. Chem. Eng. J..

[20] Tamilselvan A., Balakumar S. (2016). Anatase TiO_2_ Nanotube by Electrochemical Anodization Method: Effect of Tubes Dimension on the Supercapacitor Application. Ionics.

[21] Galstyan V., Comini E., Baratto C., Ferroni M., Poli N., Faglia G., Bontempi E., Brisotto M., Sberveglieri G. (2014). Two-phase Titania Nanotubes for Gas Sensing. Procedia Eng..

[22] Adán C., Marugán J., Sánchez E., Pablos C., van Grieken R. (2016). Understanding the Effect of Morphology on the Photocatalytic Activity of TiO_2_ Nanotube Array Electrodes. Electrochim. Acta.

[23] Sreekantan S., Saharudin K.A., Wei L.C. (2011). Formation of TiO_2_ Nanotubes via Anodization and Potential Applications for Photocatalysts, Biomedical Materials, and Photoelectrochemical cell. IOP Conf. Ser. Mater. Sci. Eng..

[24] Nguyen Q.A., Bhargava Y.V., Devine T.M. (2008). Titania Nanotube Formation in Chloride and Bromide Containing Electrolytes. Electrochem. Commun..

[25] Allam N.K., Shankar K., Grimes C.A. (2008). Photoelectrochemical and Water Photoelectrolysis Properties of Ordered TiO_2_ Nanotubes Fabricated by Ti Anodization in Fluoride-Free HCl Electrolytes. J. Mater. Chem..

[26] Taib M.A.A., Majnis M.F., Berahim M. (2018). Formation of Titanium Dioxide Nanoparticles by Anodization of Valve Metals. J. Prog. Energy Environ..

[27] Hahn R., Macak J.M., Schmuki P. (2007). Rapid Anodic Growth of TiO_2_ and WO_3_ Nanotubes in Fluoride Free Electrolytes. Electrochem. Commun..

[28] Hahn R., Lee H., Kim D., Narayanan S., Berger S., Schmuki P. (2008). Self-Organized Anodic TiO_2_-Nanotubes in Fluoride Free Electrolytes. ECS Trans..

[29] Lee K., Mazare A., Schmuki P. (2014). One-Dimensional Titanium Dioxide Nanomaterials: Nanotubes. Chem. Rev..

[30] Chiarello G.L., Ferri D., Selli E. (2011). Effect of the CH_3_OH/H_2_O Ratio on the Mechanism of the Gas-Phase Photocatalytic Reforming of Methanol on Noble Metal-Modified TiO_2_. J. Catal..

[31] Ye Y., Feng Y., Bruning H., Yntema D., Rijnaarts H.H.M. (2018). Photocatalytic Degradation of Metoprolol by TiO_2_ Nanotube Arrays and UV-LED: Effects of Catalyst Properties, Operational Parameters, Commonly Present Water Constituents, and Photo-Induced Reactive Species. Appl. Catal. B Environ..

[32] Hurum D.C., Agrios A.G., Gray K.A., Rajh T., Thurnauer M.C. (2003). Explaining the Enhanced Photocatalytic Activity of Degussa P25 Mixed-Phase TiO_2_ Using EPR. J. Phys. Chem. B.

[33] Bickley R.I., Gonzalez-Carreno T., Lees J.S., Palmisano L., Tilley R.J.D. (1991). A Structural Investigation of Titanium Dioxide Photocatalysts. J. Solid State Chem..

[34] Jaihindh D.P., Fu Y.P. (2017). Facile Synthesis of Deep Eutectic Solvent Assisted BiOCl/BiVO_4_@AgNWs Plasmonic Photocatalysts Under Visible Light Enhanced Catalytic Performance. Catal. Today.

[35] Shaabani A., Afshari R. (2018). Magnetic Ugi-Functionalized Graphene Oxide Complexed with Copper Nanoparticles: Efficient Catalyst Toward Ullman Coupling Reaction in Deep Eutectic Solvents. J. Colloid Interface Sci..

[36] Khandelwal S., Tailor Y.K., Kumar M. (2016). Deep Eutectic Solvents (DESs) as Eco-Friendly and Sustainable Solvent/Catalyst Systems in Organic Transformations. J. Mol. Liq..

[37] Li G., Liu Z.Q., Lu J., Wang L., Zhang Z. (2009). Effect of Calcination Temperature on the Morphology and Surface Properties of TiO_2_ Nanotube Arrays. Appl. Surf. Sci..

[38] Yu J., Wang B. (2010). Effect of Calcination Temperature on Morphology and Photoelectrochemical Properties of Anodized Titanium Dioxide Nanotube Arrays. Appl. Catal. B Environ..

[39] Ng S., Yam F.K., Beh K., Hassan Z. (2014). Titanium Dioxide Nanotubes in Chloride Based Electrolyte: An Alternative to Fluoride Based Electrolyte. Sains Malays..

[40] Bervian A., Coser E., Khan S., Pianaro S.A., Aguzzoli C., Marcuzzo J.S., Baldan M.R., Malfatti C.D.F. (2017). Evolution of TiO_2_ Nanotubular Morphology Obtained in Ethylene Glycol/Glycerol Mixture and its Photoelectrochemical Performance. Mater. Res..

[41] Regonini D., Bowen C.R., Jaroenworaluck A., Stevens R. (2013). A Review of Growth Mechanism, Structure and Crystallinity of Anodized TiO_2_ Nanotubes. Mater. Sci. Eng. R Rep..

[42] Mohammadpour F., Moradi M. (2015). Double-layer TiO_2_ Nanotube Arrays by Two-Step Anodization: Used in Back and Front-Side Illuminated Dye-Sensitized Solar Cells. Mater. Sci. Semicond. Proc..

[43] Giorgi L., Dikonimos T., Giorgi R., Buonocore F., Faggio G., Messina G., Lisi N. (2018). Electrochemical Synthesis of Self-Organized TiO_2_ Crystalline Nanotubes without Annealing. Nanotechnology.

[44] Wang J., Li H., Sun Y., Bai B., Zhang Y., Fan Y. (2016). Anodization of Highly Ordered TiO_2_ Nanotube Arrays Using Orthogonal Design and Its Wettability. Int. J. Electrochem. Sci..

[45] Chen S., Xiao Y., Wang Y., Hu Z., Zhao H., Xie W. (2018). A Facile Approach to Prepare Black TiO_2_ with Oxygen Vacancy for Enhancing Photocatalytic Activity. Nanomaterials.

[46] Zhu Q., Peng Y., Lin L., Fan C.M., Gao G.Q., Wang R.X., Xu A.W. (2014). Stable Blue TiO_2−x_ Nanoparticles for Efficient Visible Light Photocatalysts. J. Mater. Chem. A.

[47] Liu F., Lu L., Xiao P., He H., Qiao L., Zhang Y. (2012). Effect of Oxygen Vacancies on Photocatalytic Efficiency of TiO_2_ Effect of Oxygen Vacancies on Photocatalytic Efficiency of TiO_2_ Nanotubes Aggregation. Bull. Korean Chem. Soc..

[48] Antony R., Mathews T., Dasgupta A., Sitaram D., Tyagi A.K., Raj B. (2011). Rapid Breakdown Anodization Technique for the Synthesis of High Aspect Ratio and High Surface Area Anatase TiO_2_ Nanotube Powders. J. Solid State Chem..

[49] De M.L., Laciste M.T., Tolosa N.C., Lu M.C. (2018). Effect of Catalyst Calcination Temperature in the Visible Light Photocatalytic Oxidation of Gaseous Formaldehyde by Multi-Element Doped Titanium Dioxide. Environ. Sci. Pollut. Res..

[50] Park G.C., Seo T.Y., Park C.H., Lim J.H., Joo J. (2017). Effects of Calcination Temperature on Morphology, Microstructure, and Photocatalytic Performance of TiO_2_ Mesocrystals. Ind. Eng. Chem. Res..

[51] Bashiri R., Mohamed N.M., Kait C.F., Sufian S., Khatani M. (2017). Enhancing Photoelectrochemical Hydrogen Production Over Cu and Ni Doped Titania Thin Film: Effect of Calcination Duration. J. Environ. Chem. Eng..

[52] Zhang W., Yang B., Chen J. (2012). Effects of Calcination Temperature on Preparation of Boron-Doped TiO_2_ by Sol-Gel Method. Int. J. Photoenergy.

[53] Pishkar N., Jedi-soltanabadi Z., Ghoranneviss M. (2018). Reduction in the Band Gap of Anodic TiO_2_ Nanotube Arrays by H_2_ Plasma Treatment. Results Phys..

[54] Mioduska J., Zielińska-Jurek A., Janczarek M., Hupka J. (2016). The Effect of Calcination Temperature on Structure and Photocatalytic Properties of WO_3_/TiO_2_ Nanocomposites. J. Nanomater..

[55] Al-Hada N.M., Mohamed Kamari H., Baqer A., Shaari A., Saion E. (2018). Thermal Calcination-Based Production of SnO_2_ Nanopowder: An Analysis of SnO_2_ Nanoparticle Characteristics and Antibacterial Activities. Nanomaterials.

[56] Dhanalakshmi J., Iyyapushpam S., Nishanthi S.T., Malligavathy M., Padiyan D.P. (2017). Investigation of Oxygen Vacancies in Ce Coupled TiO_2_ Nanocomposites by Raman and PL Spectra. Adv. Nat. Sci. Nanosci. Nanotechnol..

[57] Ghows N., Entezari M.H. (2010). Ultrasound with Low Intensity Assisted the Synthesis of Nanocrystalline TiO_2_ Without Calcination. Ultrason. Sonochem..

[58] Yu J.G., Yu H.G., Cheng B., Zhao X.J., Yu J.C., Ho W.K. (2003). The Effect of Calcination Temperature on the Surface Microstructure and Photocatalytic Activity of TiO_2_ Thin Films Prepared by Liquid Phase Deposition. J. Phys. Chem. B.

[59] Sang N.X., Huong P.T.L., Thy T.T.M., Dat P.T., Minh V.C., Tho N.H. (2018). Crystalline Deformation and Photoluminescence of Titanium Dioxide Nanotubes During in Situ Hybridization with Graphene: An Example of the Heterogeneous Photocatalyst. Superlattices Microstruct..

[60] Cheong Y.L., Yam F.K., Ng S., Hassan Z., Ng S.S., Low I. (2015). Fabrication of titanium dioxide nanotubes in fluoride-free electrolyte via rapid breakdown anodization. J. Porous Mater..

[61] Varghese O.K., Gong D., Paulose M., Grimes C.A., Dickey E.C. (2003). Crystallization and High-Temperature Structural Stability of Titanium Oxide Nanotube Arrays. J. Mater. Res..

[62] Low I.M., Albetran H., Prida V.M., Vega V., Manurung P., Ionescu M. (2013). A Comparative Study on Crystallization Behavior, Phase Stability, and Binding Energy in Pure and Cr-doped TiO_2_ Nanotubes. J. Mater. Res..

[63] Kogo K., Yoneyama H., Tamura H. (1980). Photocatalytic oxidation of cyanide on platinized titanium dioxide. J. Phys. Chem..

[64] Zhang Y., Chen J., Li X. (2010). Preparation and Photocatalytic Performance of Anatase/Rutile Mixed-Phase TiO_2_ Nanotubes. Catal. Lett..

[65] He J., Du Y., Bai Y., An J., Cai X., Chen Y., Wang P., Yang X., Feng Q. (2019). Facile Formation of Anatase/Rutile TiO_2_ Nanocomposites with Enhanced Photocatalytic Activity. Molecules.

[66] Lair A., Ferronato C., Chovelon J.M., Herrmann J.M. (2008). Naphthalene Degradation in Water by Heterogeneous Photocatalysis: An Investigation of the Influence of Inorganic Anions. J. Photochem. Photobiol. A.

[67] Yang L., Liu Z., Shi J., Zhang Y., Hu H., Shangguan W. (2007). Degradation of Indoor Gaseous Formaldehyde by Hybrid VUV and TiO_2_/UV processes. Sep. Purif. Technol..

[68] Escobedo Salas S., Serrano Rosales B., de Lasa H. (2013). Quantum Yield with Platinum Modified TiO_2_ Photocatalyst for Hydrogen Production. Appl. Catal. B Environ..

[69] Laciste M.T., de Luna M.D.G., Tolosa N.C., Lu M.C. (2017). Degradation of Gaseous Formaldehyde via Visible Light Photocatalysis using Multi-Element Doped Titania Nanoparticles. Chemosphere.

[70] Sheng C., Wang C., Wang H., Jin C., Sun Q., Li S. (2017). Self-Photodegradation of Formaldehyde Under Visible-Light by Solid Wood Modified via Nanostructured Fe-Doped WO_3_ Accompanied with Superior Dimensional Stability. J. Hazard..

